# Role of Na^+^-K^+^ ATPase Alterations in the Development of Heart Failure

**DOI:** 10.3390/ijms251910807

**Published:** 2024-10-08

**Authors:** Naranjan S. Dhalla, Vijayan Elimban, Adriana Duris Adameova

**Affiliations:** 1 Institute of Cardiovascular Sciences, St. Boniface Hospital Albrechtsen Research Centre, Winnipeg, MB R2H 2A6, Canada; velimban@sbrc.ca; 2 Department of Physiology and Pathophysiology, Max Rady College of Medicine, University of Manitoba, Winnipeg, MB R3E 0J9, Canada; 3 Department of Pharmacology and Toxicology, Faculty of Pharmacy, Comenius University, 83232 Bratislava, Slovakia; adriana.duris.adameova@uniba.sk

**Keywords:** heart failure, cardiac Na^+^-K^+^ ATPase, myocardial cation content, phospholemman phosphorylation, endogenous steroids, cardiac dysfunction, Na^+^-K^+^ ATPase isoforms

## Abstract

Na^+^-K^+^ ATPase is an integral component of cardiac sarcolemma and consists of three major subunits, namely the α-subunit with three isoforms (α_1_, α_2_, and α_3_), β-subunit with two isoforms (β_1_ and β_2_) and γ-subunit (phospholemman). This enzyme has been demonstrated to transport three Na and two K ions to generate a trans-membrane gradient, maintain cation homeostasis in cardiomyocytes and participate in regulating contractile force development. Na^+^-K^+^ ATPase serves as a receptor for both exogenous and endogenous cardiotonic glycosides and steroids, and a signal transducer for modifying myocardial metabolism as well as cellular survival and death. In addition, Na^+^-K^+^ ATPase is regulated by different hormones through the phosphorylation/dephosphorylation of phospholemman, which is tightly bound to this enzyme. The activity of Na^+^-K^+^ ATPase has been reported to be increased, unaltered and depressed in failing hearts depending upon the type and stage of heart failure as well as the association/disassociation of phospholemman and binding with endogenous cardiotonic steroids, namely endogenous ouabain and marinobufagenin. Increased Na^+^-K^+^ ATPase activity in association with a depressed level of intracellular Na^+^ in failing hearts is considered to decrease intracellular Ca^2+^ and serve as an adaptive mechanism for maintaining cardiac function. The slight to moderate depression of Na^+^-K^+^ ATPase by cardiac glycosides in association with an increased level of Na^+^ in cardiomyocytes is known to produce beneficial effects in failing hearts. On the other hand, markedly reduced Na^+^-K^+^ ATPase activity associated with an increased level of intracellular Na^+^ in failing hearts has been demonstrated to result in an intracellular Ca^2+^ overload, the occurrence of cardiac arrhythmias and depression in cardiac function during the development of heart failure. Furthermore, the status of Na^+^-K^+^ ATPase activity in heart failure is determined by changes in isoform subunits of the enzyme, the development of oxidative stress, intracellular Ca^2+^-overload, protease activation, the activity of inflammatory cytokines and sarcolemmal lipid composition. Evidence has been presented to show that marked alterations in myocardial cations cannot be explained exclusively on the basis of sarcolemma alterations, as other Ca^2+^ channels, cation transporters and exchangers may be involved in this event. A marked reduction in Na^+^-K^+^ ATPase activity due to a shift in its isoform subunits in association with intracellular Ca^2+^-overload, cardiac energy depletion, increased membrane permeability, Ca^2+^-handling abnormalities and damage to myocardial ultrastructure appear to be involved in the progression of heart failure.

## 1. Introduction

Since the discovery of Na^+^-K^+^ ATPase by JC Skou in 1957, numerous investigators have examined the physiological, biochemical and pharmacological aspects of this enzyme to establish its role in cardiac function, health and disease [1,2,3,4,5,6,7,8,9,10,11,12,13,14,15]. Na^+^-K^+^ ATPase is embedded in the sarcolemmal membrane and its main function is to transport three Na and two K ions through an energy-dependent mechanism for the generation of an electrochemical gradient across the cell membrane and the maintenance of cation homeostasis in cardiomyocytes. This enzyme serves as a receptor for exogenous cardiac glycosides such as digoxin and ouabain; different cardiotonic steroids, including endogenous ouabain and marinobufagenin, are known to modulate cardiac contractile activity upon its inhibition. The regulatory role of Na^+^-K^+^ ATPase with respect to the structure–function relationship and pathological implications, as well as its therapeutic aspects, has also been studied extensively [16,17,18,19,20,21,22,23,24,25,26,27,28,29,30]. Na^+^-K^+^ ATPase has been shown to act as a signal transducer, regulating different metabolic processes in cardiomyocytes as well as cellular growth, differentiation, survival and death. The activity of Na^+^-K^+^ ATPase is regulated by different hormones through the phosphorylation/dephosphorylation of phospholemman (FXYD1), which is considered to be a γ-subunit of this enzyme. Na^+^-K^+^ ATPase is known to be made up of two other subunits, namely the α-subunit and β-subunit; the α-subunit occurs in three isoforms (α_1_, α_2_ and α_3_) whereas the β-subunit has two isoforms (β_1_ and β_2_) in the heart. These three subunits (α, β and γ) of this enzyme have been demonstrated to play specific roles in determining the function of Na^+^-K^+^ ATPase.

While the activation of Na^+^-K^+^ ATPase is considered to be involved in cell survival as an adaptive mechanism, the marked inhibition of this enzyme was reported to induce arrhythmias, cellular damage, cell death and cardiac dysfunction. Furthermore, low doses of cardiac glycosides, which moderately inhibit Na^+^-K^+^ ATPase, are known to exert positive inotropic action and beneficial effects, whereas high doses of these agents, which produce a marked inhibition of Na^+^-K^+^ ATPase, are known to exert cardiotoxic actions during heart failure. Thus, in view of the conflicting reports regarding alterations in the Na^+^-K^+^ ATPase activities in different types of failing hearts, the exact role of this enzyme in cardiac dysfunction during the development of heart failure is not understood [31,32,33]. This article therefore aims to review the existing literature regarding the involvement of changes in the status of this enzyme in the pathogenesis of heart failure. In addition, it plans to describe the association of alterations in Na^+^-K^+^ ATPase activity with changes in different isoforms of this enzyme in failing hearts. Efforts will be made to determine the involvement of some signal transduction mechanisms in modifying the regulation of Na^+^-K^+^ ATPase in different experimental models of heart failure. The relationship among changes in Na^+^-K^+^ ATPase activity, myocardial Na^+^, and K^+^, Mg^2+^ and Ca^2+^ content, as well as contractile activity during the progression of heart failure, will also be examined to gain insight into the multifunctional and complex nature of this enzyme.

## 2. Status of Na^+^-K^+^ ATPase Activity and Isoenzymes

### 2.1. Alterations in Na^+^-K^+^ ATPase Activity in Failing Hearts

Several conflicting reports concerning changes in the activity of Na^+^-K^+^ ATPase in several types of heart failure have appeared in the literature [31,32,33,34,35,36,37,38,39,40,41,42,43,44,45]. Some of the studies showing increased, unaltered and decreased Na^+^-K^+^ ATPase activities [33,34,35,36,37,38,39,40,41,42,43,44,45] in both experimental and clinical conditions of heart failure are shown in Table 1. An increase in Na^+^-K^+^ ATPase activity was seen in the BIO 14.6 strain of cardiomyopathic hamsters with a moderate degree of congestive heart failure, as well as in heart failure in dogs following mitral insufficiency or pressure overload due to aortic banding [34,35,36]. On the other hand, heart failure due to pulmonary stenosis in dogs, as well as aortic insufficiency or aortic constriction in rabbits, were not associated with any changes in Na^+^-K^+^ ATPase activity [33,37]. While the observations of no changes in Na^+^-K^+^ ATPase activity in failing hearts may be due to their being observations of early stages of heart failure, increased Na^+^-K^+^ ATPase activity has also been reported in cardiac dysfunction due to vitamin deficiency in rats [46], K^+^-deficiency in guinea pigs [47] or the administration of cobalt in rats [48]. An increase in the activity of Na^+^-K^+^ ATPase was suggested to reduce the intracellular concentration of Na^+^, decrease the entry of Ca^2+^ through the Na^+^-Ca^2+^ exchange system, reduce the intracellular concentration of Ca^2+^ and thus depress cardiac function [35,36]. In this regard, it is noteworthy that the treatment of heart failure due to pressure overload with digoxin or prazosin was found to improve cardiac function and reduce the increased Na^+^-K^+^ ATPase activity [49,50]. Furthermore, the treatment of heart failure due to mitral insufficiency with prazosin was also observed to prevent the deterioration of cardiac function as well as the increased Na^+^-K^+^ ATPase activity in the failing heart [51]. Since an increase in Na^+^-K^+^ ATPase activity can be seen to reduce the concentration of intracellular Ca^2+^ and suppress the occurrence of intracellular Ca^2+^-overload, it is suggested that the increased Na^+^-K^+^ ATPase activity in the failing heart may serve as an adaptive mechanism for the induction of beneficial effects.

The involvement of Na^+^-K^+^ ATPase in the pathogenesis of heart failure was suggested on the basis of various studies showing a reduction in the activity of this enzyme in failing hearts [32,38,39,40,41,42,43,44,45]. From the information in Table 1, it may be noted that depressions in Na^+^-K^+^ ATPase activity were observed in failing hearts in rabbits due to aortic constriction, in genetic cardiomyopathy in hamsters (UM-X7.1) and in tachycardia-induced cardiomyopathy in pigs [38,39,40]. Furthermore, heart failure due to pulmonary constriction in dogs, as well as myocardial infarction in rats, showed depressed Na^+^-K^+^ ATPase activity [41,42]. Low Na^+^-K^+^ ATPase activity was also found to occur in human hearts as a consequence of end-stage dilated cardiomyopathy, congestive heart failure and idiopathic dilated cardiomyopathy [43,44,45]. Biopsies taken from failing hearts during cardiac transplantation revealed depressed levels of Na^+^-K^+^ ATPase activity [32,52]. A reduction in Na^+^-K^+^ ATPase activity was also reported in animals with hypoadrenalism, as well as in contractile failure in isolated rat hearts perfused with hypoxic or substrate-free medium [53,54,55]. It is pointed out that improvements in cardiac function following heart failure were associated with attenuation of the depressed Na^+^-K^+^ ATPase activity in heart failure in infarcted animals treated with a metabolic inhibitor, propionyl L-carnitine [56]. These observations indicate that a reduction in Na^+^-K^+^ ATPase activity in cardiomyocytes may be involved in the pathogenesis of cardiac dysfunction in heart failure.

To gain further information regarding the role of alterations in Na^+^-K^+^ ATPase activity in cardiac dysfunction, the left-ventricle sarcolemmal ATPase activities were monitored at different stages of heart failure due to myocardial infarction in rats [57]. The results presented in Table 2 indicate that cardiac dysfunction, as reflected by a significant increase in LVEDP and depressions in both +dP/dT and −dP/dT, at early stages of heart failure was not associated with any significant alteration in Na^+^-K^+^ ATPase activity. On the other hand, cardiac dysfunction at both moderate and severe stages of heart failure was accompanied by marked depressions in Na^+^-K^+^ ATPase activity. Since sarcolemmal Mg^2+^-ATPase activity decreased significantly at early, moderate and severe stages of heart failure due to myocardial infarction (Table 2), it is likely that the observed reduction in Na^+^-K^+^ ATPase activity at moderate and severe stages of heart failure may be due to a generalized defect in sarcolemma. In another set of experiments [58], a marked depression of Na^+^-K^+^ ATPase activity at moderate stages of heart failure was not associated with any significant decrease in Mg^2+^-ATPase activity (Table 3). Furthermore, the treatment of myocardial infarcted animals with enalapril, an angiotensin-converting enzyme inhibitor, or losartan, an angiotensin II receptor antagonist, were observed to improve cardiac function and attenuate the depression in Na^+^-K^+^ ATPase activity without any changes in Mg^2+^-ATPase activity (Table 3). Changes in Na^+^-K^+^ ATPase activity were also examined in the UM-X7.1 strain of cardiomyopathic hamsters of different age groups [59]. On the basis of the accumulation of abdominal fluid (ascites), as well as increases in lung weight (wt), liver wt and heart to body wt ratio, these cardiomyopathic hamsters, at 90–100 days, 120–160 days, 160–200 days and 200–280 days, were considered to be at pre-failure, early failure, moderate failure and severe failure stages, respectively [59]. It can be seen from Table 4 that Na^+^-K^+^ ATPase activity was depressed at pre-failure as well as at all stages of heart failure in the UM-X7.1 strain of cardiomyopathic hamsters without any significant alterations in Mg^2+^-ATPase activity except in animals at the severe stage of heart failure. These observations provide further support to the view that reduced Na^+^-K^+^ ATPase activity may be specifically involved not only in the development but also in the progression of heart failure.

### 2.2. Alterations in Na^+^-K^+^ ATPase Isoenzymes in Failing Hearts

Extensive studies in both humans and animals revealed that Na^+^-K^+^ ATPase in failing hearts is composed of the α-, β- and γ-subunits, which determine its overall activity and are known to have distinctly different functions from each other [7,9,17,20,31,60,61,62]. While the α-subunit primarily contains binding sites for Na^+^, K^+^ and ATP as well as cardiotonic glycosides and plays a catalytic role in the enzyme activity, the β-subunit is concerned with the transport and anchoring of the newly synthesized Na^+^-K^+^ ATPase to the sarcolemma membrane, as well as modulation of the ATPase activity. On the other hand, the γ-subunit or phospholemman regulates Na^+^-K^+^ ATPase activity upon phosphorylation/dephosphorylation because unphosphorylated and phosphorylated forms of phospholemman exert inhibitory and stimulatory actions, respectively, on Na^+^-K^+^ ATPase. Decreased Na^+^-K^+^ ATPase activity in human heart failure was found to be associated with lower protein expressions for the α_1_-, α_3_- and β_1_-subunits without any changes in their respective mRNA levels in cardiomyocytes [63,64]. No alterations in protein or mRNA levels for the α_2_-subunit of Na^+^-K^+^ ATPase were observed in human heart failure [63,64]. The downregulation of Na^+^-K^+^ ATPase density in patients with dilated cardiomyopathy was also not associated with any change in mRNA levels for the α_1_-, α_2_ and α_3_-subunits [65].

Since Na^+^-K^+^ ATPase α-subunit isoforms have a similar affinity for cardiac glycosides, it was suggested that the increased sensitivity that occurs during heart failure to cardiac glycosides is probably due to a depression in Na^+^-K^+^ ATPase density rather than to the selective inhibition of any α-subunit isoform of the enzyme [66]. It should also be pointed out that while reduced Na^+^-K^+^ ATPase activity in heart failure due to tachycardia, aortic valve disease, hypertension and different cardiomyopathies [67,68] is accompanied by depressed levels of the α_1_-, α_3_- and β_1_-subunits, the overexpression of the α_2_-subunit of Na^+^-K^+^ ATPase, which has been shown to be involved in Ca^2+^-signaling [69], was observed to attenuate cardiac hypertrophy and heart failure [70,71]. In addition, the α_2_-subunit has been shown to protect against cardiac remodeling and β-adrenoreceptor desensitization after myocardial infarction [72]. In contrast, β_1_-subunit knock-out mice were insensitive to the positive inotropic effect of ouabain [73]. Depression in the phosphorylation of phospholemman (γ-subunit) has also been reported to be associated with decreased Na^+^-K^+^ ATPase activity in heart failure [9,21,31]. Thus, different isoforms of Na^+^-K^+^ ATPase have been demonstrated to be involved in diverse pathological functions during the development of heart failure.

Because there is an increase in the plasma level of marinobufagenin and a depression in the sensitivity of Na^+^-K^+^ ATPase to this cardiotonic steroid, as well as a reduction in the α_1_-isoform during cardiac hypertrophy and heart failure, it was suggested that a shift in endogenous Na^+^-K^+^ ATPase ligand production is linked to a shift in myocardial Na^+^-K^+^ ATPase isoforms [74]. While the level of α_1_-isoform was lower in decompensated cardiac hypertrophy than that in compensated cardiac hypertrophy in guinea pigs due to aortic stenosis, increased levels of the α_2_-isoform were involved in decompensated hypertrophy and the development of arrhythmias [75]. Heart failure induced by tachycardia in dogs decreased Na^+^-K^+^ ATPase activity, α_3_-isoform protein and α_3_-isoform mRNA levels without any changes in α_1_- or α_2_-isoform proteins. These alterations in the failing heart were found to be similar to those seen upon chronic infusion of norepinephrine, except that the downregulation of the α_3_-isoform protein was not associated with a decrease in the α_3_-isoform mRNA levels [76,77]. The information in Table 5 indicates that the reduction in Na^+^-K^+^ ATPase activity in the UM-X7.1 strain of cardiomyopathic hamsters at a severe stage of heart failure was shown to be due to increased levels of α_1_- and β_1_-isoform proteins, but mRNA levels for the α_2_-subunit were decreased [78]. It can also be seen from Table 5 that the protein levels of the α_3_-isoform were depressed in these cardiomyopathic hamsters; however, the mRNA levels for this subunit were not detectable in either control or diseased animals [78]. The reduced Na^+^-K^+^ ATPase activity in the MS 200 strain of cardiomyopathic hamsters with dilated cardiomyopathy was accompanied by decreased levels of α_1_- and β_1_-isoform proteins without any changes in the level of the α_2_-subunit [79]. It was also pointed out that a reduction in Na^+^-K^+^ ATPase activity, α_1_-subunit protein content and α_1_-subunit mRNA levels was also observed in the BIO14.6 strain of hamsters with severe congestive heart failure [80,81].

**Figure 1:**
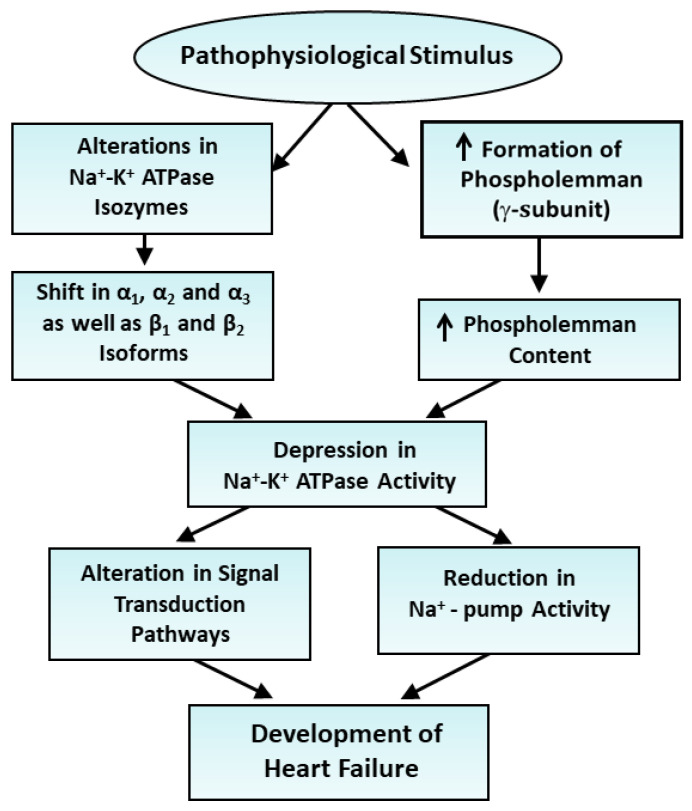
Role of alterations in Na^+^-K^+^ ATPase isozymes and depression in the enzyme activity in the development of heart failure. ↑—increase.

From Table 6, it can be observed that the reduced level of Na^+^-K^+^ ATPase activity in a rat model of heart failure due to myocardial infarction was associated with a dramatic depression in both protein and mRNA levels for the α_1_-, α_2_- and β_1_-subunits as well as an increase in both protein and mRNA levels for the α_3_-subunits; these alterations were partially prevented upon the treatment of the animals with imidapril, an angiotensin II-converting inhibitor [58]. In another study, the treatment of infarcted animals with a moderate degree of heart failure with a blocker of the renin–angiotensin system such as enalapril and losartan was observed to attenuate the decreased protein and mRNA levels in the α_2_-isoform, as well as the increased protein and mRNA levels in the α_3_-isoform in the failing heart following 8 weeks of myocardial infarction [82]. End-stage heart failure due to 40 weeks of myocardial infarction was associated with reduced Na^+^-K^+^ ATPase activity and mRNA levels in the α_2_-isoform, as well as increased mRNA levels in the α_1_- and α_3_-isoforms; these alterations were also partially attenuated by treatment with imidapril [83]. It should be mentioned that the reduced Na^+^-K^+^ ATPase activity in heart failure due to myocardial infarction was also reported to be associated with the decreased expression of protein and mRNA levels for the α_2_-subunit and increased expression of the α_2_-subunit, without any changes in the levels of α_1_- and β_1_-subunits of Na^+^-K^+^ ATPase [84]. These observations suggest that cardiac dysfunction in failing hearts is associated with reduced Na^+^-K^+^ ATPase activity and a wide variety of alterations in the α_1_-, α_2_-, α_3_- and β_1_-subunits of Na^+^-K^+^ ATPase depending upon the type, species and stage of heart failure. The summary of observations regarding the role of alterations in Na^+^-K^+^ ATPase isozymes in depressing the enzyme activity in heart failure is illustrated in Figure 1.

## 3. Signal Transduction and Regulation of Na^+^-K^+^ ATPase in Heart Failure

In addition to serving as a Na^+^-pump, Na^+^-K^+^ ATPase has been reported to function as a signal transducer upon inhibition by both exogenous and endogenous cardiotonic steroids [15,85,86,87,88,89,90]. The inhibition of Na^+^-K^+^ ATPase by cardiac glycosides indicates that the Ras/Raf/MEK/MAPK pathway is activated upstream of Ras by tyrosine kinase Src. Furthermore, these agents stimulate redox-activated protein kinases such as protein kinase A (PKA), protein kinase C (PKC) and calcium–calmodulin kinase II (CaMKII) [85,86,87,88,89,90]. The activated kinases are intimately involved in signal transduction mechanisms which are associated with the increased formation of oxyradicals, enhanced oxidative phosphorylation and impaired mitochondrial Ca^2+^ retention in both normal and failing hearts [88,89,90,91,92]. It should be emphasized that an increase in the intracellular concentration of Na^+^ achieved via the inhibition of this enzyme with cardiac glycosides not only promotes the elevation of intracellular Ca^2+^ through the Na^+^-Ca^2+^ exchange system but also promotes the activation of sarcolemmal L-type Ca^2+^ channels, and store-operated Ca^2+^-channels also help to increase the intracellular Ca^2+^ [93,94]. Furthermore, an increased concentration of intracellular Na^+^ results in the release of Ca^2+^ from the sarcoplasmic reticulum, involving CaMKII, PKA and inositol-3-phosphate receptor systems [95]. The signal transduction mechanisms initiated upon the inhibition of Na^+^-K^+^ ATPase have also been reported to induce cell growth, cardiac hypertrophy heart failure and arrhythmias [96,97,98]. In addition, several cardiotonic steroids, upon increases in the intracellular concentrations of both Na^+^ and Ca^2+^, have been observed to develop oxidative stress, apoptosis and cardiomyocyte injury due to the activation of diverse signal transduction systems [99,100,101,102,103].

The activity of Na^+^-K^+^ ATPase in normal and diseased hearts is regulated by different hormones, prostaglandins, growth factors and endogenous ligands through the participation of various signal transduction mechanisms [7,8,9,18,19,22,23,104]. The activation of β_1_-adrenergic receptors by catecholamines has been shown to affect the cyclic AMP-PKA mechanism, whereas the activation of β_2_- and β_3_-adrenergic receptors has been reported to modify Na^+^-K^+^ ATPase activity in the failing heart through some isoform-specific mechanisms [77,105,106,107,108,109,110]. On the other hand, low concentrations of angiotensin II were reported to stimulate Na^+^-K^+^ ATPase by increasing tyrosine kinase and MAP kinase activities, whereas high concentrations of this hormone are known to inhibit the enzyme via oxidative stress due to the marked activation of NADPH oxidase [111]. Estradiol and insulin-like growth factor have been shown to increase the expression of Na^+^-K^+^ ATPase isoforms due to the formation of nitric oxide [23,112]. Thromboxane β_2_, a product of prostaglandin, was shown to depress Na^+^-K^+^ ATPase activity [113]. Likewise, endogenous ligand marinobufagenin have been observed to decrease Na^+^-K^+^ ATPase activity by affecting the α_1_-subunit of the enzyme [7,8,18,114]. Thus, a wide variety of hormones and other factors produced during the development of heart failure have been reported to modify Na^+^-K^+^ ATPase activity in cardiomyocytes.

It is now well established that the regulation of Na^+^-K^+^ ATPase is mainly carried out as a consequence of the phosphorylation of phospholemman through different protein kinases as well as dephosphorylation by protein phosphatase-1 in normal and failing hearts [5,20,21,61,62,115,116,117,118,119,120]. It should be noted that unphosphorylated phospholemman exerts an inhibitory effect on Na^+^-K^+^ ATPase, whereas phosphorylated phospholemman increases the Na^+^-K^+^ ATPase activity due to changes in the protein conformation. Alterations in both the expression and phosphorylation of phospholemman have been reported to contribute to maladaptive cardiac hypertrophy, reduced Na^+^-K^+^ ATPase activity, arrhythmias and heart failure [121,122,123]. The activation of Na^+^-K^+^ ATPase by phosphorylated phospholemman is isoform-specific and has been shown to provide cardioprotection in diseased heart [124]. Stabilizing the enzyme via the antibody SSA412 at a specific site of this enzyme was shown to activate Na^+^-K^+^ ATPase and produce an inotropic effect [125]. A polyclonal antibody (DRRSAb), which was Na^+^-K^+^ ATPase DR region-specific, was also found to activate the enzyme and produce cardioprotection against ischemic injury through the stimulation of extracellular signal-regulated kinase and the phosphoinositide 3-kinase/Akt pathway [126]. Furthermore, this antibody was shown to improve cardiac function, alleviate cardiac hypertrophy and reduce oxidative stress by stabilizing membrane Na^+^-K^+^ ATPase in isoproterenol-treated mice [127]. Accordingly, it has been suggested that the activation as well as the stabilization of Na^+^-K^+^ ATPase may result in a new strategy for improving the therapy of heart failure.

A decrease in Na^+^-K^+^ ATPase activity was observed during the transition of cardiac hypertrophy to heart failure; this change was associated with an increase in the plasma level of marinobufagenin, a decrease in α_1_-isoform and an increase in the α_3_-isoform of the enzyme [7,8,18,74]. In fact, the infusion of marinobufagenin was also shown to depress Na^+^-K^+^ ATPase activity, reduce α_1_-isoform and induce heart failure [103]. Different mineralocorticoids such as aldosterone, as well as various inflammatory cytokines such as the tumor necrosis factor (TNF-α), the levels of which are increased in heart failure, have been reported to reduce Na^+^-K^+^ ATPase expression as well as activity and produce cardiac fibrosis [128,129,130,131,132]. Furthermore, oxidative stress, intracellular Ca^2+^-overload, the activation of proteases and alterations in the lipid composition of the sarcolemmal membrane have been demonstrated to depress Na^+^-K^+^ ATPase activity in heart failure [27,28,31,133]. Observations regarding the regulatory effects of diverse factors provide evidence that a reduction in Na^+^-K^+^ ATPase activity may be involved in the pathogenesis of heart failure. On the other hand, various hypertrophic stimuli, which are known to be associated with adaptive mechanisms, were shown to increase Na^+^-K^+^ ATPase activity as well as gene expression [31,134]. Thus, it appears that interventions that may induce an increase in Na^+^-K^+^ ATPase activity may prove beneficial in therapy aiming to treat heart failure.

**Figure 2:**
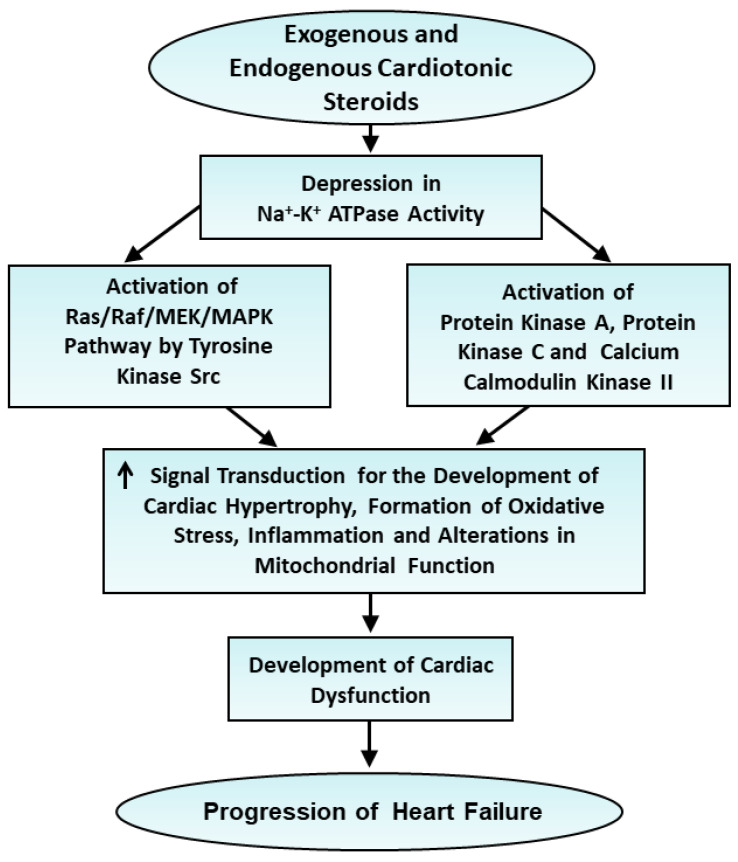
Role of changes in signal transduction mechanisms following depression of Na^+^-K^+^ ATPase activity in the progression of heart failure. ↑—increase.

From this discussion, it is evident that the activation of the signal transduction pathway as a consequence of a depression in Na^+^-K^+^ ATPase is a distinct function independent of its Na^+^-pump activity. The signal-transducing function of Na^+^-K^+^ ATPase was discovered by Zijian Xie in 2002 [89]. This view is supported by the fact that low doses of cardiac glycosides were observed to affect the signal transduction pathway without any effect on Na^+^ pump activity. The salient features of the involvement of signal transduction pathways in the development of heart failure are shown in Figure 2.

## 4. Alterations in Na^+^-K^+^ ATPase and Intracellular Cation Content in Contractile Dysfunction

In view of the important role of some cations, such as Na^+^, K^+^, Ca^2+^ and Mg^2+^, in cardiac function and metabolism, different subcellular organelles, including sarcolemma, sarcoplasmic reticulum and mitochondria, are known to be intimately involved in regulating the intracellular concentration of these cations. However, these regulatory membrane systems become defective following heart failure and lead to marked alterations in the electrolyte composition of the myocardium during the development of cardiac dysfunction in various types of failing hearts [135,136]. The high levels of intracellular Na^+^ and low levels of intracellular K^+^ in the failing heart are generally attributed to reduced Na^+^-K^+^ ATPase activity, whereas the increased levels of intracellular Ca^2+^ are attributed to the augmentation of Na^+^-Ca^2+^ exchange activity. However, several investigators have indicated that high levels of Na^+^ in the failing myocardium may also be a consequence of the increased Na^+^-H^+^ exchange systems in both the sarcolemma and mitochondria, as well as the increased activation of sarcolemmal Na^+^-channels [137,138,139,140,141,142,143]. Furthermore, the elevated levels of Ca^2+^ in the failing heart are considered to be due to the increased release of Ca^2+^ from the sarcoplasmic reticulum, as well as depressed Ca^2+^-pump activity in the sarcolemmal membrane. Thus, the involvement of the reduced activity of Na^+^-K^+^ ATPase and elevated levels of intracellular Na^+^ and Ca^2+^ cannot be considered to fully explain the pathogenesis of heart failure.Although heart failure is a complex problem and there are very many factors which may play different roles in this devastating disease, the following discussion is centered around gaining some information regarding the relationship among changes in the Na^+^-K^+^ ATPase activity, alterations in myocardial cation content and contractile dysfunction under various experimental conditions. In a rabbit model of cardiac hypertrophy and heart failure induced by a catheter with or without bacterial infection [144,145,146], it was observed that contractile dysfunction in hypertrophied failing hearts was associated with depressed Na^+^-K^+^ ATPase activity, an increased intracellular concentration of Na^+^ and a decreased level of intracellular K^+^ (Table 7). However, the intracellular Ca^2+^ in the hypertrophid failing heart was decreased and alterations in the intracellular Mg^2+^ were variable (Table 7). 

Although the observed changes in the concentrations of Na^+^, K^+^ and Mg^2+^ in this model of cardiac hypertrophy and failure are in agreement with the results for other types of failing hearts [38,39,40,41,42,43,44,45,147], the decrease in the concentration of intracellular Ca^2+^ in these experimental hearts was different from the other results. In this regard, it is pointed out that heart failure has been shown to be associated with either Ca^2+^ overload or Ca^2+^ deficiency [136]. We also examined the relationship between changes in cation content and Na^+^-K^+^ ATPase activity using isolated rat hearts perfused with different interventions known to induce contractile dysfunction [135]. The perfusion of hearts with Ca^2+^-free medium, which is known to generate no contractile activity [135], was found to decrease all cation content as well as Na^+^-K^+^ ATPase activity (Table 8). In contrast, reperfusion of the Ca^2+^-free perfused hearts with Ca^2+^-containing medium [135] was observed to increase Na^+^ and Ca^2+^ content in addition to depressing Na^+^-K^+^ ATPase activity, as well as K^+^ and Mg^2+^ content (Table 8).

**Figure 3:**
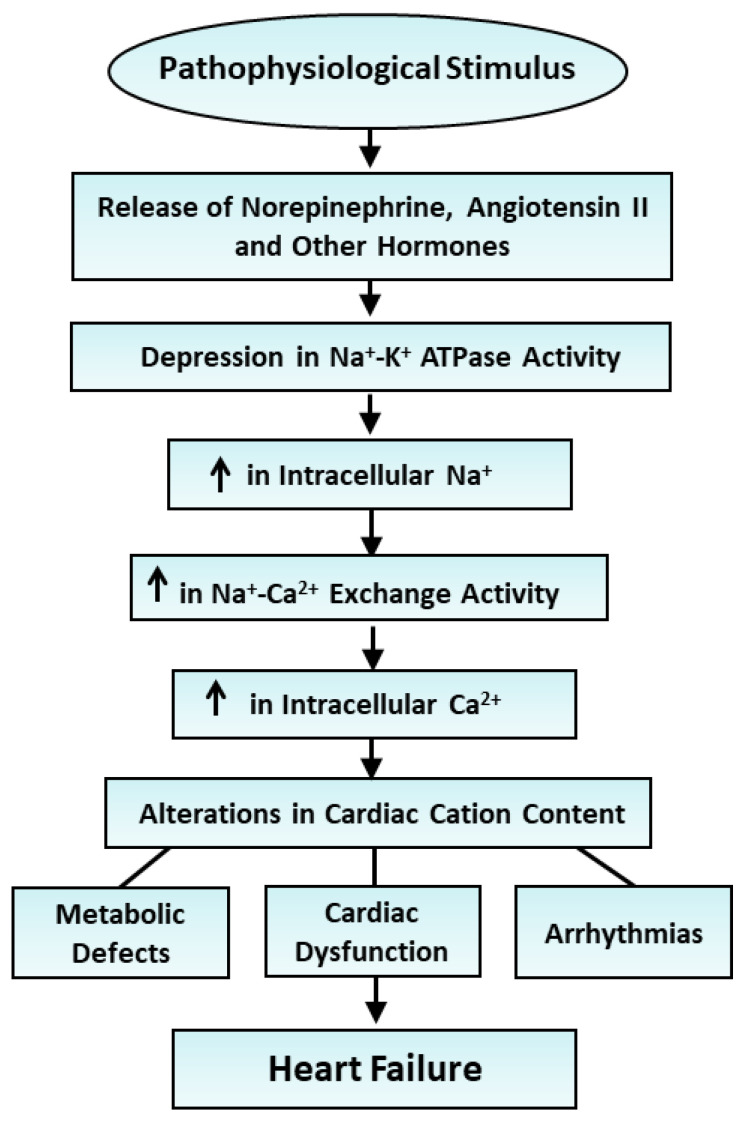
Role of various hormones in the depression Na^+^-K^+^ ATPase activity and changes in cardiac cation contents in the development of heart failure. ↑—increase.

The inability of the heart to generate contractile activity upon perfusion for 20 min with Na^+^-free or K^+^-free medium [135] was associated with a depression in Na^+^-K^+^ ATPase activity (Table 9). However, myocardial Na^+^, K^+^ and Mg^2+^ contents were decreased and Ca^2+^ level was increased upon perfusion with the Na^+^-free medium, whereas Na^+^ and Ca^2+^ contents were increased and K^+^ as well as Mg^2+^ contents were decreased upon perfusion with the K^+^-free medium (Table 9). The perfusion of hearts with substrate-free medium for 2 h resulted in loss of contractile function [135], a marked reduction in Na^+^-K^+^ ATPase activity and a decrease in K^+^ and Mg^2+^ content, in addition to an increase in Na^+^ content, without any changes in Ca^2+^ levels (Table 10). Although hearts perfused with hypoxic medium for 30 min showed no contractile function [135] and a depression in Na^+^-K^+^ ATPase activity, myocardial Na^+^ content was increased whereas K^+^, Ca^2+^ and Mg^2+^ contents were decreased (Table 10). These observations provide evidence that this reduced Na^+^-K^+^ ATPase activity may be associated with the development of cardiac dysfunction but showed no relationship with changes in myocardial cation content. It appears that alterations in cation content in the failing heart are determined by diverse mechanisms including changes in Na^+^-K^+^ ATPase activity, sarcolemmal and mitochondrial Na^+^-H^+^ exchange activities, sarcolemmal and sarcoplasmic reticulum cation channels’ activities, and sarcolemmal permeability. Thus, alterations in Na^+^ and Ca^2+^ contents in the failing heart cannot be considered to be exclusively due to the reduction in Na^+^-K^+^ ATPase expression or activity in the failing heart. The ways in which Na^+^-K^+^ ATPase induces alterations in cardiac cation content and subsequent changes in metabolic processes, as well as changes in the pathogenesis of heart failure and associated arrhythmias and cardiac dysfunction, are depicted in Figure 3.

## 5. Conclusions

Extensive studies have indicated that Na^+^-K^+^ ATPase not only acts as a Na^+^-pump for maintaining myocardial cation composition but also serves as a signal transducer, modifying cardiac function and metabolism, when its activity is inhibited by different cardiotonic glycosides and steroids. Different isoforms of the α- and β-subunits of Na^+^-K^+^ ATPase are involved in the catalytic function and in anchoring this enzyme to the sarcolemmal membrane, respectively, whereas the γ-subunit (phospholemman) of Na^+^-K^+^ ATPase, by virtue of its phosphorylation/dephosphorylation ability, is known to regulate the enzyme activity. Several hormones and growth factors have been shown to regulate Na^+^-K^+^ ATPase activity by affecting different redox-sensitive or redox-insensitive tyrosine kinases in an isoform-specific manner in both normal and failing hearts. Some endogenous ligands, such as marinobufagenin, the levels of which are increased in heart failure, are known to have an inhibitory affect on Na^+^-K^+^ ATPase activity. Some interventions, such as oxidative stress and inflammatory cytokines, the levels of which are increased in heart failure, have been shown to adversely affect Na^+^-K^+^ ATPase activity as well as inducing the development of apoptosis and fibrosis. However, in spite of the numerous associations between alterations in Na^+^-K^+^ ATPase activity and a wide variety of defects in metabolic, cation transport and functional processes, as well as Ca^2+^-handling abnormalities, the exact role that changes in Na^+^-K^+^ ATPase activity play in the pathogenesis of heart failure remains far from clear.

In this article, we reviewed the literature regarding alterations in Na^+^-K^+^ ATPase activity during the development of heart failure. It was observed that Na^+^-K^+^ ATPase activity is increased, decreased or unaltered in the failing heart in both experimental and clinical situations. These variable changes in Na^+^-K^+^ ATPase activity appear to depend upon the stage and type of pathological stimuli responsible for inducing heart failure. Nonetheless, some studies have shown that increased Na^+^-K^+^ ATPase activity in the failing heart may promote the efflux of Na^+^, depress the intracellular concentration of Na^+^ and thereby reduce the intracellular level of Ca^2+^ due to its lack of effect on the Na^+^-Ca^2+^ exchange system. Accordingly, the increased Na^+^-K^+^ ATPase activity due to heart failure may serve as an adaptive mechanism. On the other hand, the reduced activity of Na^+^-K^+^ ATPase in the failing heart has been reported to depress the removal of Na^+^ from cardiomyocytes, increase the intracellular concentration of Na^+^ and thus increase the intracellular concentration of Ca^2+^ due to the participation of the Na^+^-Ca^2+^ exchange system. In view of the harmful effects of an excessive amount of intracellular Ca^2+^ on cardiac function and metabolism, the reduced Na^+^-K^+^ ATPase activity in the failing heart is considered to be a pathogenic factor for the development and/or progression of heart failure. It is therefore of critical importance that some novel therapies are developed to increase Na^+^-K^+^ ATPase activity in the failing heart to improve the treatment of heart failure. This view is supported by the observation that some specific Na^+^-K^+^ ATPase antibody preparations, which stabilize and activate Na^+^-K^+^ ATPase, have been reported to improve cardiac function and prevent arrhythmias in heart failure.

## Figures and Tables

**Table 1:** Alterations of Na^+^-K^+^ ATPase activity in different types of failing hearts.

Na^+^-K^+^ ATPase Activities in Different Types of Failing Hearts	References
**A. Increased Na^+^-K^+^ ATPase Activity**	
Cardiomyopathic hamsters (BI0 14.6)	Sulakhe and Dhalla, 1973 [34]
Mitral insufficiency in dogs	Khatter and Prasad, 1976 [35]
Pressure overload in dogs (aortic banding)	Prasad et al., 1979 [36]
**B. Unaltered Na^+^-K^+^ ATPase activity**	
Pulmonary stenosis in dogs	Mead et al, 1971 [37]
Aortic insufficiency in rabbits	Despa et al., 2002 [33]
Aortic constriction in rabbits	Despa et al., 2002 [33]
**C. Decreased Na^+^-K^+^ ATPase activity**	
Aortic constriction in rabbits	Yazako and Fujii, 1972 [38]
Cardiomyopathic hamsters (UM-X7.1)	Dhalla et al. 1976 [39]
Tachycardia induced cardiomyopathy in pigs	Spinale et al, 1992 [40]
Pulmonary constriction in dogs	Fan et al, 1993 [41]
Myocardial infarction in rats	Dixon et al, 1992 [42]
Dilated cardiomyopathy in human	Norgaard et al. 1990 [43]
Congestive heart failure in human	Ellingsen et al, 1994 [44]
Idiopathic dilated cardiomyopathy in humans	Ishino et al, 1999 [45]

**Table 2:** Alterations in cardiac function and sarcolemmal Na^+^-K^+^ ATPase at different stages of heart failure due to myocardial infarction in rats.

Parameters	Control	Different Stages of Heart Failure		
		Early Failure	Moderate Failure	Severe Failure
**A. Cardiac function**				
LV systolic pressure (LVSP; mmHg)	143 ± 9.6	130 ± 11.3	128 ± 10.5	92 ± 7.4 *
LV end-diastolic pressure (LVEDP; mmHg)	2.6 ± 0.6	12.4 ± 1.8 *	13.6 ± 2.1 *	14.4 ± 1.5 *
Rate of cardiac contraction (+dP/dt; mmHg/s)	5680 ± 240	4228 ± 302 *	4200 ± 320 *	2718 ± 306 *
Rate of cardiac relaxation (−dP/dt; mmHg/s)	5148 ± 225	3126 ± 240 *	3228 ± 236 *	2342 ± 300 *
**B. ATPase activity**				
Mg^2+^ ATPase (μmol Pi/mg/h)	86.6 ± 3.2	71.5 ± 3.2 *	58.3 ± 3.2 *	55.6 ± 3.3 *
Na^+^-K^+^ ATPase (μmol Pi/mg/h)	26.2 ± 2.8	24.4 ± 2.3	15.2 ± 1.8 *	13.0 ± 1.2 *

Each value is a mean ± SE of 8 experiments. The data are based on the results in our article [57]. LV, left ventricle; *—*p* < 0.05 vs. respective control value. Animals at 4, 8 and 16 weeks after myocardial infarction were designated at early, moderate and severe stages of heart failure, respectively.

**Table 3:** Improvement of cardiac dysfunction and depressed sarcolemmal Na^+^-K^+^ ATPase activity in heart failure upon treatment of infarcted rats with enalapril (10 mg/kg/day) or losartan (20 mg/kg/day).

Parameters	Control	Heart Failure due to Myocardial Infarction		
		Untreated	Enalapril	Losartan
**A. Cardiac function**				
LVSP (mm Hg)	133 ± 4.9	128 ± 3.2	131 ± 3.8	127 ± 3.9
LVEDP (mm Hg)	4.0 ± 0.2	15.9 ± 1.3 *	7.5 ± 0.6 ^#^	6.9 ± 0.5 ^#^
+dP/ dt (mm Hg/s)	9208 ± 1075	4800 ± 745 *	7690 ± 680 ^#^	7544 ± 722 ^#^
−dP/ dt (mm Hg/s)	8788 ± 956	4326 ± 590 *	7248 ± 702 ^#^	7312 ± 690 ^#^
**B. ATPase activities**				
Mg^2+^ ATPase (μmol Pi/mg/h)	93.6 ± 6.9	81.2 ± 7.4	85.5 ± 6.2	86.9 ± 7.8
Na^+^-K^+^ ATPase (μmol/mg/h)	22.1 ± 0.82	12.9 ± 0.84 *	18.3 ± 0.59 ^#^	18.6 ± 0.84 ^#^

Three weeks after the induction of myocardial infarction, the animals were treated with or without enalapril, or losartan for 4 weeks. The data are based on results from our paper [58]. Values are mean ± SE of 6 animals in each group. LVSP—left ventricle systolic pressure; LVEDP—left ventricle diastolic pressure; *—*p* < 0.05 vs. control; #—*p* < 0.05 vs. untreated.

**Table 4:** General characteristics and alterations in sarcolemmal Na^+^-K^+^ ATPase activity of cardiomyopathic hamsters (UM-X 7.1) of different age groups.

	Animals at Different Age Groups			
Parameters	90–100 Days	120–160 Days	160–200 Days	200–280 Days
**A. General characteristics**				
Ascites (mL)	ND	1.3 ± 0.42 *	2.9 ± 0.31 *	7.8 ± 0.84 *
Lung wt (% increase)	0.5 ± 0.34	5.4 ± 0.49 *	26.2 ± 1.7 *	35.2 ± 1.9 *
Liver wt (% increase)	1.3 ± 0.69	1.2 ± 0.58	14.5 ± 0.86 *	41.2 ± 2.13 *
Heart/body wt ratio (% increase)	8.9 ± 0.4 *	15.6 ± 0.8 *	22.5 ± 1.7 *	24.6 ± 1.5 *
**B. ATPase activities**				
Mg^2+^ ATPase (μmol/Pi/mg/h)				
Control	21.2 ± 0.9	22.8 ± 1.7	21.4 ± 1.5	21.9 ± 2.1
Cardiomyopathic	20.5 ± 0.7	21.4 ± 2.3	20.0 ± 1.7	14.2 ± 1.4 *
Na^+^-K^+^ ATPase (μmol/Pi/mg/h)				
Control	4.6 ± 0.5	5.8 ± 0.6	6.7 ± 0.4	7.5 ± 0.7
Cardiomyopathic	2.4 ± 0.6 *	3.2 ± 0.5 *	3.8 ± 0.2 *	3.6 ± 0.9 *

On the basis of general characteristics, cardiomyopathic hamsters of different age groups were identified at different stages of heart failure. ND, non-detectable; *—*p* < 0.05 vs. respective control value. The data are based on results from our article [59]. Each value is a mean of ± SE of 4 experiments in each group. Changes in lung wt, liver wt and heart/body wt ratio are expressed as % of value in the control animals of same age group. Cardiomyopathic animals of 90–100, 120–160, 160–200 and 200–280 days age groups were at pre failure, early failure, moderate failure and severe failure stages, respectively.

**Table 5:** Alterations in sarcolemmal Na^+^-K^+^ activity, isoform protein content and isoform mRNA levels in 250 days old cardiomyopathic hamsters (UM-X7.1) at severe stages of heart failure.

Parameters	Control	Cardiomyopathic
**A. ATPase activities**		
Mg^2+^ ATPase (μmol Pi/mg/h)	30.5 ± 2.6	28.2 ± 1.5
Na^+^-K^+^ ATPase (μmol Pi/mg/h)	8.9 ± 1.2	5.4 ± 1.0 *
**B. Na^+^-K^+^ ATPase subunit**		
**protein content** (% control)		
α_1_-subunit	100	164 ± 27 *
α_2_-subunit	100	82 ± 6 *
α_3_-subunit	100	69 ± 11 *
β_1_-subunit	100	146 ± 22 *
**B. Na^+^-K^+^ ATPase activities**		
**mRNA levels** (% of control)		
α_1_-subunit	100	165 ± 20 *
α_2_-subunit	100	60 ± 12 *
α_3_-subunit	ND	ND
β_1_-subunit	100	151 ± 14 *

Values are mean ± SE of 3 experiments. The data are based on results from our article [78]. The band for α_3_ mRNA level was not detectable. Changes in protein content and in RNA level were expressed as % of control animals of same age group. *, *p* < 0.05 vs. respective control value; ND, not detectable.

**Table 6:** Alterations in sarcolemmal Na^+^-K^+^ ATPase activity, subunit protein content and subunit mRNA levels in heart failure due to myocardial infarction with or without Imidapril (1 mg/Kg/day).

		Heart Failure due to Myocardial Infarction	
Parameters	Control	Untreated	Imidapril
**A. ATPase activity**			
Mg^2+^ ATPase (μmol Pi/mg/h)	92.4 ± 8.6	85.0 ± 6.8	86.4 ± 9.2
Na^+^-K^+^ ATPase (μmol Pi/mg/h)	22.6 ± 2.7	8.9 ± 3.2 *	18.4 ± 1.6 ^#^
**B. Na^+^-K^+^ ATPase subunit**			
**protein content (%)**			
α_1_-subunit	100	17.4 ± 1.8 *	40.4 ± 2.9 ^#^
α_2_-subunit	100	10.5 ± 1.2 *	42.6 ± 3.8 ^#^
α_3_-subunit	100	160 ± 7.5 *	104 ± 2.9 ^#^
β_1_-subunit	100	65 ± 5.9 *	82 ± 4.3 ^#^
C. **Na^+^-K^+^ ATPase subunit**			
**mRNA level** (%)			
α_1_-subunit	100	55 ± 6.2 *	84 ± 5.1 ^#^
α_2_-subunit	100	46 ± 7.5 *	96 ± 5.8 ^#^
α_3_-subunit	100	247 ± 12.2 *	113 ± 8.6 ^#^
β_1_-subunit	100	48 ± 7.1 *	78 ± 8.2 ^#^

Three weeks after the induction of myocardial infarction, the animals were treated with or without imidapril, and ACE inhibitor. The data are based on results from our paper [58]. Values are mean ± SE of 5 to 6 animals in each group. *, *p* < 0.05 vs. control; #, *p* < 0.05 vs. untreated.

**Table 7:** Alterations in cardiac function, cation content and Na^+^-K^+^ ATPase activity at different times of inducing bacterial endocarditis in rabbits.

Parameters	Control	Uninfected Catheterized		Infected Catheterized	
		3 Days	6 Days	3 Days	6 Days
**A. Cardiac function**					
Left heart wt/body					
wt (×10^3^) ratio	1.34 ± 0.04	1.61 ± 0.12 *	2.06 ± 0.8 *	1.82 ± 0.09 *	2.65± 0.11 *
Heart rate (beats/min)	259 ± 7.2	286 ± 4.5 *	302 ± 5.6 *	292 ± 6.1 *	203 ± 5.9 *
+dP/dt (mmHg/s)	476 ± 26	456 ± 22	404 ± 28 *	259 ± 31 *	198 ± 24 *
−dP/dt (mmHg/s)	332 ± 21	320 ± 20	326 ± 33	217 ± 22 *	185 ± 27 *
**B. Cation content**					
Sodium (μmol/g heart dry wt)	118 ± 8.4	129 ± 105	214 ± 12.1 *	166 ± 10.9 *	246 ± 13.2 *
Potassium (μmol/g heart dry wt)	336 ± 12.1	318 ± 13.6	262 ± 13.0 *	282 ± 14.2 *	248 ± 14.6 *
Calcium (μmol/g heart dry wt)	7.4 ± 0.25	6.4 ± 0.28*	4.2 ± 0.33 *	5.5 ± 0.28 *	5.6 ± 0.26 *
Magnesium (μmol/g heart dry wt)	31.8 ± 0.97	N.D.	33.7 ± 1.16	28.5 ± 0.58 *	35.1 ± 0.72 *
**C. ATPase activity**					
Mg^2+^ ATPase (μmol Pi/mg/h)	19.4 ± 1.3	18.1 ± 0.9	15.9 ± 0.6 *	16.0 ± 0.7 *	13.8 ± 0.8 *
Na^+^-K^+^ ATPase (μmol Pi/mg/h)	7.6 ± 0.4	6.4 ± 0.4	5.5 ± 0.4 *	4.2 ± 0.3 *	3.4 ± 0.3 *

Endocarditis was induced in catheterized animals by injecting 10^7^ organisms/ kg of Streptococcus viridans for 3 and 6 days. Uninfected catheterized animals were used for comparison whereas sham operated animals served as control. The data are based on results from our papers [144,145,146]. Each value is mean ± SE of 4 to 6 animals. *—*p* < 0.05 vs respective control value; +dP/dt, rate of contraction; −dP/dt, rate of relaxation. N.D., Not determined.

**Table 8:** Changes in sarcolemmal Na^+^-K^+^ ATPase activity and cation content in isolated rat hearts subjected to perfusion with Ca^2+^-free medium for 20 min or reperfusion for 10 min with medium containing 1.25 mM Ca^2+^.

Parameters	Control	Perfusion with Ca^2+^-Free Medium	Reperfusion with Ca^2+^-Containing Medium after Perfusion with Ca^2+^-Free Medium
**A. ATPase activity**			
Mg^2+^ ATPase (μmol Pi/mg/h)	25.0 ± 1.8	19.1 ± 1.2 *	14.1 ± 2.0 *
Na^+^-K^+^ ATPase (μmol Pi/mg/h)	11.7 ± 0.5	7.5 ± 0.8 *	3.4 ± 0.5 *
**B. Cation content**			
(μmol/wet wt)			
Sodium	22.6 ± 1.7	11.2 ± 0.7 *	37.8 ± 3.0 *
Potassium	54.7 ± 1.3	30.5 ± 1.5 *	22.0 ± 2.0 *
Calcium	1.9 ± 0.1	0.8 ± 0.1 *	3.1 ± 0.1 *
Magnesium	13.7 ± 0.1	10.7 ± 0.2 *	5.8 ± 0.1 *

Hearts subjected to perfusion with Ca^2+^-free medium or reperfusion with normal medium did not generate any contractile activity. The data are based on results from our article [135]. Values are mean ± SE of 6 to 8 hearts in each group. *, *p* < 0.05 vs. control.

**Table 9:** Changes in sarcolemmal Na^+^-K^+^ ATPase activity and cation content of isolated rat hearts perfused with sodium-free or potassium-free medium for 20 min.

Parameters	Control	Sodium-Free Perfusion for 20 min	Potassium-Free Perfusion for 30 min
**A. ATPase activity**			
Mg^2+^ ATPase (μmol Pi/mg/h)	25.8 ± 2.1	30.1 ± 1.8	23.5 ± 2.0
Na^+^-K^+^ ATPase (μmol Pi/mg/h)	11.2 ± 1.0	6.3 ± 0.6	7.7 ± 0.3 *
**B. Cation content**			
(μmol/g wet wt)			
Sodium	24.9 ± 1.4	12.1 ± 0.3 *	47.8 ± 1.6 *
Potassium	53.5 ± 1.5	41.9 ± 3.0 *	31.9 ± 1.3 *
Calcium	1.9 ± 0.1	3.2 ± 0.1 *	3.8 ± 0.2 *
Magnesium	14.5 ± 0.1	13.9 ± 0.2 *	13.3 ± 0.1 *

Hearts perfused with sodium-free or potassium-free medium for 20 min did not generate any contractile activity. The data are based on results from our article [135]. Values are mean ± SE of 6 to 8 hearts in each group. *, *p* < 0.05 vs. control.

**Table 10:** Changes in sarcolemmal Na^+^-K^+^ ATPase activity and cation content of isolated rat hearts upon perfusion with substrate-free or hypoxia medium.

Parameters	Control	Substrate Free (2 h Perfusion)	Hypoxia (30 min Perfusion)
**A. ATPase activity**			
Mg^2+^ ATPase (μmol Pi/mg/h)	24.0 ± 1.7	28.8 ± 1.9 *	28.3 ± 2.2 *
Na^+^-K^+^ ATPase (μmol Pi/mg/h)	12.8 ± 1.1	5.9 ± 0.8 *	6.2 ± 0.04 *
**B. Cation content**			
(μmol/g wet wt)			
Sodium	26.9 ± 1.2	67.3 ± 2.1 *	58.3 ± 1.9 *
Potassium	53.1 ± 1.2	31.7 ± 1.5 *	43.5 ± 1.3 *
Calcium	1.9 ± 0.1	1.8 ± 0.1	1.3 ± 0.2 *
Magnesium	13.9 ± 0.1	12.4 ± 0.2 *	12.4 ± 0.2 *

Hearts perfused with substrate-free medium for 2 h or with hypoxic medium for 30 min failed to generate any contractile activity. The data are based on results from our article [135]. Values are mean ± SE of 6 to 8 hearts in each group. *, *p* < 0.05 vs. control.
